# Exploring the intersection of adverse childhood experiences, pediatric chronic pain, and rheumatic disease

**DOI:** 10.1186/s12969-022-00674-x

**Published:** 2022-02-14

**Authors:** Maitry Sonagra, Jeremy Jones, Mackenzie McGill, Sabrina Gmuca

**Affiliations:** 1 Division of Rheumatology, Department of Pediatrics, Children’s Hospital of Philadelphia, Roberts Center for Pediatric Research, Roberts Center for Pediatric Research, Philadelphia, PA USA; 2 PolicyLab, Children’s Hospital of Philadelphia, Philadelphia, PA USA; 3 Center for Pediatric Clinical Effectiveness, Children’s Hospital of Philadelphia, Philadelphia, PA USA; 4 Heart Center, Center for Integrative Brain Research, Seattle Children’s Hospital, Seattle, WA USA; 5 Perelman School of Medicine, University of Pennsylvania, Philadelphia, PA USA; 6 Division of General Pediatrics, Department of Pediatrics, Children’s Hospital of Philadelphia, Philadelphia, PA USA

**Keywords:** Adverse childhood experiences, Pediatric rheumatology, Pediatric chronic pain

## Abstract

**Background:**

While the general relationship between ACEs and the development of chronic pain has become increasingly clear, how ACEs may shape a child’s clinical presentation with regards to chronic pain has yet to be fully expounded. We aimed to determine the association between ACEs and clinical manifestations of pediatric chronic pain and explore the interaction of ACEs and pediatric rheumatic disease among youth with chronic pain on health-related outcomes.

**Methods:**

We conducted a cross-sectional cohort study of patients aged ≤18 years with chronic pain seen in a pediatric rheumatology amplified pain clinic between August 2018 and July 2020. We stratified subjects into three groups: no ACEs, one ACE, and ≥ 2 ACEs. We assessed clinical signs and symptoms associated with the presence of ACEs using Chi-square or Wilcoxon-rank test. The association between ACEs as well as other variables of interest with functional impairment was tested using simple and multivariable linear regression.

**Results:**

Of the 412 patients included, more than 75% of patients reported at least one ACE. Most frequent included history of mental illness in a first degree relative (56%) and parental divorce or separation (20%). Those with ≥2 ACEs had more somatic symptoms, worse functional disability, and a higher proportion of mental health conditions. There appeared to be a dose dependent interaction between ACEs and functional disability from co-morbid rheumatologic disease. In multivariable regression, higher verbal pain score, symptom severity score (SSS), and presence of autonomic changes were associated with estimated average increase in FDI score (β = 1.05, 1.95 and 4.76 respectively; all *p* < 0.01).

**Conclusion:**

Children with chronic pain and/or rheumatologic diseases who are exposed to ACEs are at increased risk of greater symptomatology, functional disability, and somatization of symptoms. Our findings indicate an ongoing need for systemic evaluation of ACEs in children with chronic pain and/or rheumatic disease and incorporation of trauma-based care.

## Background

Chronic pain is common in the pediatric population, affecting approximately one-quarter of children and adolescents [1–4]. Further, approximately 5% of youth report moderate to severe chronic pain, accompanied by impaired physical and psychosocial functioning [1, 2]. While current understanding of the development of chronic pain is limited, previous studies have shown a clear link between psychosocial stressors and chronic pain [5]. Chronic stressors, in particular, are thought to play a driving role in the development of chronic pain through biological mechanisms such as central sensitization, a state of increased neuronal activity within the central nervous system (CNS) that amplifies nociceptive signaling [6]. These stressors also cause sustained activation of the hypothalamic-pituitary-adrenal (HPA) axis, leading to excess cortisol production and, over time, dysregulation of the body’s sympathetic and parasympathetic systems [7, 8]. The interplay of these complex factors has led to the development of the integrative biopsychosocial model which suggests that biologic (genetic, neurobiological, neuroendocrine), psychologic (subjective experience of events, coping abilities), and social (peer and family environment, trauma, social learning) factors all are important aspects in shaping how a child experiences pain that must be considered by the pediatric heath care provider [9].

A specific class of stressors, Adverse Childhood Experiences (ACEs), have been linked with poorer physical and mental health and, more specifically, higher rates of pain and functional disability in pediatric patients [10]. ACEs encompass a broad category of experiences including abuse (physical, sexual, and emotional) and neglect, as well as other chronic stressors such as parental mental illness, financial insecurity, substance use, divorce, incarceration, and domestic violence. A large cross-sectional analysis of the 2016–2017 National Survey of Children’s Health (NSCH) found that 70% of children with chronic pain had experienced at least one ACE during their lifetime, compared to 48% of children without chronic pain [10]. Furthermore, this relationship between ACEs and chronic complex medical conditions is dose dependent. For example, rates of chronic headaches are strongly associated with the number of ACEs experienced, and Rubinstein et al. showed that a higher ACE score was associated with an increased likelihood of having a chronic medical condition [11, 12]. This relationship was even stronger for the development of arthritis, suggesting an especially strong link between chronic stress and rheumatologic disease, concordant with previously defined relationships between stress and autoimmunity [11, 13]. While these relationships have previously been described in adult literature, this was the first pediatrics study to directly link ACEs, childhood arthritis, and chronic pain [14]. However, these findings were by parental report and clinical data were not available to further elucidate variations in clinical findings.

**Fig. 1:**
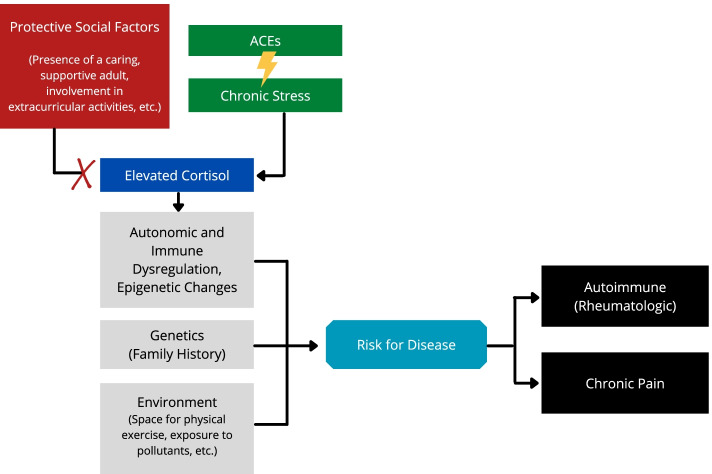
Chronic pain syndromes and rheumatologic disease share overlapping driving factors. The etiologies of chronic pain and rheumatologic disease are complex and manifold. However, genetic predisposition, environmental factors, and chronic stress are all relevant. This figure proposes a model for how ACEs may play an underappreciated (but addressable) role in pathogenesis for both disease processes through neuroendocrine changes

Better understanding of these relationships is vital, as optimal prevention or treatment strategies may be dictated by the impact of specific ACEs within a biopsychosocial context. To this end, we propose a conceptual model built upon previous work highlighting how ACEs, chronic pain, and rheumatologic disease may all be interrelated and guiding our hypothesis development (Fig. 1) [15–18]. These drivers are quite common and, given the high prevalence of chronic pain and its lasting impact on physical, social, and emotional well-being well into adulthood, the relationship between ACEs and chronic pain is of significant interest not just for pain specialists, but all pediatric providers [19].

Our team is uniquely positioned to examine the role of ACEs in pediatric chronic pain via a large cohort of patients seen in the Center for Amplified Musculoskeletal Pain Clinic (pediatric rheumatology subspeciality pain clinic) at The Children’s Hospital of Philadelphia. We aimed to describe the relationship between the presence of ACEs and pain severity, clinical characteristics, and functional disabiltiy among a chronic pain population. We hypothesized that the presence of at least once ACE was associated with greater pain and greater pain-related physical disability and psychosocial impairment. We also aimed to explore if children with chronic pain and ACEs had a greater likelihood of also having a co-morbid rheumatologic condition [18]. We hypothesized those with ACE exposure were more likely to have a co-morbid rheumatologic condition and that ACE exposure would be associated with increased functional disability due to rheumatologic disease when present.

## Methods

### Study population

We performed a retrospective cross-sectional cohort study of patients ≤18 years old with chronic pain presenting to a pediatric rheumatology pain clinic at a tertiary care hospital from August 2018 to July 2020, who were enrolled in an existing IRB approved registry database. Each patient, upon initial presentation to the clinic, received a thorough evaluation by both a physician and a clinical psychologist. This prospective patient registry houses data from patients’ initial clinic evaluation and includes about 96% of all new patients. It is periodically updated and maintained to include variables of interest in a standardized process utilizing the password secure RedCap (Research Electronic Data Capture) database. Research assistants in the Division of Rheumatology’ Rheumatology Research Core (RRC) are responsible for ensuring data entry is completed, cleaned, and updated appropriately. Patients from this IRB-approved registry were included in the present study if they met the following criteria: 1) diagnosed with chronic pain by the treating physician and 2) initial clinic intake conducted between August 2018 and July 2020. This study received exemption from the study site’s Institutional Review Board.

### Data collection

Patient data included demographics and clinical characteristics abstracted from initial visit chart review providing data on age, race, ethnicity, sex, self-reported past medical, psychological and surgical history, past medications, family mental health history, and educational information. All clinic visits included information regarding physical exam findings, current medications, pain severity and quality, and diagnosis and treatment recommendations. Additionally, all visits included Patient Reported Outcomes (PROs). Patients completed a self-report measure of functional disability called the Functional Disability Inventory (FDI; ranging from 0 to 60 with greater scores indicating greater functional impairment), which was our main study outcome. The FDI is an accepted, validated, and frequently used self-report measure of limitations in function among youth with chronic pain [20]. The FDI score can also be categorized into levels of functional disability as follows: minimal to no disability (0–12); mild disability [13–20]; moderate [21–29]; and severe (≥30) [20, 21]. Other PROs included verbal pain report (0–10); symptom severity score (SSS; 0–12 withgreater scores indicate a greater number of somatic symptoms and fatigue); and widespread pain index (WPI; 0–19) with greater numbers indicating more body parts involved) according to the American College of Rheumatology 2010 criteria for fibromyalgia syndrome [22–24].

Of interest for the present study was the presence and number of adverse childhood experiences (ACEs) abstracted from patient’s medical chart; including the psychologist’s evaluation note from the initial clinic visit. For this study, history of any of the following events were categorized as an ACE as defined in our prior work and based on established survey measures for exposure to ACEs [25, 26]: 1) verbal abuse, 2) physical abuse, 3) sexual abuse, 4) parent with an alcohol problem, 5) parent with a drug problem, 6) parental divorce, 7) parental separation, 8) other household members with problems with drugs and/or alcohol, 9) household mental illness/suicide, 10) incarcerated household member, 11) economic hardship, 12) mother or stepmother is a victim of domestic violence, 13) bullying identified by the patient as having self-reported significant impact on psychosocial functioning. We also included a category for ACEs defined as “other” to include traumatic events documented in the patient’s medical record and considered to be an ACE as per findings from the original CDC-Kaiser study on ACEs and/or taking into consideration newly recognized, expanded ACEs [27, 28]. Trained clinical researchers abstracted ACEs data from the notes by the physician and psychologist from the initial clinic visit. Specifically, the researchers reviewed standardized sections of the clinic note templates including: personal relationships, psychological history, family medical/social/emotional history, presentation/mental status/behavior observations, history of present illness, patient-entered family history, life changes, etc. Although most of this information is patient-reported and some ACEs may not have been reported/documented, anything listed in the notes were abstracted and then reviewed by another member of the research team for accuracy and consistency.

Exposure to rheumatic disease was defined as a physician documented diagnosis of at least one rheumatic disease at the time of the initial clinic visit, including but not limited to uveitis, juvenile idiopathic arthritis, periodic fever syndrome, vasculitis, juvenile dermatomyositis, and Sjögren’s syndrome.

For this study, all data from the first documented clinical encounter (intake visit) were utilized. We also approximated socioeconomic status by matching patient zip codes to estimated median household income retrieved from the 2018 U.S. Census Bureau files using the web-based query portal (https://data.census.gov/cedsci/).

### Statistical analyses

Patient demographics and clinical characteristics were summarized by median and interquartile range (IQR) for continuous variables, and frequencies and percentage for categorical variable. We stratified our sample into three groups based on number of ACEs: no ACES, one ACE, and ≥ 2 ACEs. Difference in demographics, clinical characteristics and psychological issues between these groups were tested using Wilcoxon Rank Sum test for continuous variables and Chi-squared or Fisher’s exact test, as appropriate, for categorical variables. Associations between ACEs along with other variables of interest and self-reported patient-level functional disability (FDI score) were assessed using simple and multivariable linear regression. We were also interested in exploring whether the magntitude of ACEs exposure in children with chronic pain who also had a co-morbid rheumatologic condition modified the risk of functional disability, which we tested using the interaction term between number of ACEs and rheumatologic condition in our regression modeling. All analyses were completed using SAS version 9.4 (Copyright© 2002–2012 by SAS Institute Inc., Cary, NC, USA).

## Results

A total of 412 patients seen in the pediatric rheumatology pain clinic between August 2018 and July 2020 were included in the study. Table 1 shows patient characteristics. The patient population was predominantly female (83%), Caucasian (75%), and non-Hispanic (90%). The median age of patients at presentation was 14 years (IQR: 12–16). The patients presenting with ≥2 ACEs were slightly older (median age of 15; IQR: 13–16), and this trended towards a statistically significant difference between groups (*p* = 0.05). Estimated median household income of our cohort ($83,970) was markedly higher than the national median income ($65,712). However, those with two or more ACEs had significantly lower estimated median family income ($73,702; IQR: 53, 964–95, 656) than those with no ($86,167, IQR: 66,378-112,885) or only one ($89,712, IQR: 65,934-118,365) reported ACE (*p* < 0.001). A higher proportion of patients with two or more ACEs had estimated family incomes under $100,000 (81% vs 61% vs 66% in the two or more, one, and no ACEs groups respectively). Less than 1% from the ≥2 ACEs group were in the ≥150,000 income category as opposed to 7% from one ACE and 6% from the no ACEs groups (*p* < 0.01).

**Table 1:** Demographics and Clinical Characteristics among Children with Chronic Pain stratified by Adverse Childhood Experiences Exposure (*N* = 412)

	All Patients (*N* = 412)	No ACEs (*n* = 101)	1 ACE (*n* = 194)	≥2 ACEs (*n* = 117)	*P*-value
*Demographics, N (%)*					
Sex, female	342 (83%)	82 (81%)	161 (83%)	99 (85%)	0.80
Race°					
Caucasian	307 (75%)	68 (67%)	151 (78%)	88 (75%)	0.14
Black	30 (7%)	5 (5%)	11 (6%)	14 (12%)	0.07
Other	73 (18%)	28 (28%)	31 (16%)	14 (12%)	< 0.01
Ethnicity^, non-Hispanic	372 (90%)	95 (94%)	170 (88%)	107 (92%)	0.41
Age, median (IQR)	14 (12–16)	14 (11–15)	14 (12–16)	15 (13–16)	0.05
Median Household Income, median (IQR)	83,970 (62130–108,571)	86,167 (66378–112,885)	89,712 (65934–118,365)	73,702 (53964–95,656)	< 0.0001
*Pain and, Patient and Parent Reported Outcomes, N (%)*					
Verbal pain score (0–10), median (IQR)	5 (3–7)	5 (3–8	5 (3–7)	5 (3–7)	0.82
Patient FDI (0–60), median (IQR)	23 (14–33)	23 (14–32)	21 (13–32)	27 (17–34)	0.05
Parent FDI (0–60), median (IQR)	24 (13–32)	24 (12–31)	22 (13–31)	26 (18–34)	0.03
WPI (0–19)	5 (2–10)	6 (1–10)	5 (2–9)	6 (2–10)	0.27
SSS (0–12)	6 (4–8)	5 (3–8)	6 (3–8)	7 (4–9)	< 0.01
Duration of symptoms (months), median (IQR)	13 (7–36)	12 (7–24)	18 (7–39)	24 (8–48)	0.05
Autonomic Changes^o^	107 (26%)	20 (20%)	62 (32%)	25 (22%)	0.03
History of Trigger Event	115 (28%)	28 (28%)	59 (30%)	28 (24%)	0.47
Attend Traditional School	180 (44%)	51 (51%)	86 (44%)	43 (37%)	0.12
*Self-reported Cognitive and/or Psychological Conditions, N (%)*					
Anxiety / Panic attacks	234 (57%)	34 (34%)	115 (59%)	85 (73%)	< 0.0001
Depression	134 (33%)	16 (16%)	58 (30%)	60 (51%)	< 0.0001
Eating disorder	8 (2%)	0	3 (2%)	5 (4%)	0.06
Hyperactivity / ADHD	54 (13%)	7 (7%)	23 (12%)	24 (21%)	0.01
Obsessive Compulsive Disorder	38 (9%)	3 (3%)	20 (10%)	15 (13%)	0.03
Previous outpatient mental health care^±^	252 (61%)	53 (53%)	115 (59%)	84 (72%)	0.01
Previous psychiatric hospitalization	23 (6%)	2 (2%)	8 (4%)	13 (11%)	< 0.01
Suicide attempt	18 (4%)	3 (3%)	8 (4%)	7 (6%)	0.54
Suicide ideation	78 (19%)	13 (13%)	32 (17%)	33 (28%)	0.01
Co-morbid Rheumatologic condition					
At least one rheumatologic condition^^^	36 (9%)	9 (9%)	16 (8%)	11 (9%)	0.94

Abbreviations: *N* Number of subjects, *IQR* Interquartile Range, *FDI* Functional Disability Index (total scores range from 0 to 60 where higher scores indicate worse physical function), *WPI* Widespread Pain Index (total scores range from 0 to 19 where higher scores indicate more widespread pain), *SSS* Symptom Severity Score (total scores range from 0 to 12), *ADHD* Attention deficit hyperactivity disorder.

^o^Autonomic changes categories: subjects could report or demonstrate an autonomic change (including temperature change, cyanosis, edema) in > 1 category.

^±^Previous outpatient mental health care defined as seen at least once by a counselor/therapist/psychologist for pain.

^^^Rheumatologic conditions included: juvenile idiopathic arthritis (*n* = 9); enthesitis (*n* = 9); inflammatory bowel disease (*n* = 7); uveitis (*n* = 2); vasculitis (*n* = 1), chronic recurrent multifocal osteomyeltisi (*n* = 1); Lyme disease (*n* = 2); Sjögren’s syndrome (*n* = 1); other (*n* = 12).

Missing Data: Verbal Pain = 1, Patient FDI = 4, Parent FDI = 8, WPI = 9, SSS = 10, Duration = 3

Seventy-six percent of patients reported at least one ACE (Table 2). The most frequent reported ACE was a history of mental illness in a first degree relative, affecting over half (56%) of the patient population. The second most reported ACE was parental divorce or separation (20%). Other reported ACEs included abuse (7%), verbal bullying (6%), household member with a substance misuse disorder (3%), suicide attempted by a household member (3%), or a household member that went to prison (< 1%).

**Table 2:** Self- and/or Parent- Reported ACEs among Youth with Chronic Pain (*N* = 412)

ACEs	Frequency (%)
≥ 1 ACE^*^	311 (76%)
History of Mental Health Illness in first degree relative	231 (56%)
Parents Divorced/Separated	86 (20%)
Abuse - Verbal/Physical/Sexual	28 (7%)
Bullying	25 (6%)
Parent or other Household member with Alcohol or Drug problem	14 (3%)
Household member attempted suicide	14 (3%)
Household member went to prison	3 (< 1%)
Other ACEs	26 (6%)
No ACEs	101 (24%)

*Patient could report ACEs for > 1 category.

Other ACEs include: Patient’s mother or step mother is a victim of domestic violence, patient’s parents are together but never married, difficult relationship with father, patient’s mother having health issue following severe car accident, patient is adopted or foster child, patient is adopted and discovered biological brother who lives with his adopted parents and has mental health illness, patient having limited/stressful relationship with father or half siblings, loss of father, patient living with single mother and/or no contact with biological father, loss of adoptive father and substance abuse by biological mother, patient in custody issue, patient has no contact with sibling

Patients had similar pain severity and widespreadness of pain regardless of the number of ACEs experienced (Table 1). However, median FDI scores varied across the groups, with higher scores (indicating worse functional disability) among the patients with two or more ACEs by both patient-report (*p* = 0.05; Table 1) and parent-report (*p* = 0.03; Table 1). Those with higher ACEs reported more somatic symptoms with a median SSS of 7 (IQR: 4–9) in comparison to those with one ACE (median 6, IQR: 3–8) or no ACEs (median 5, IQR: 3–8; *p* < 0.01). Similarly, those with two or more ACEs were more likely to have comorbid self- or parent-reported mental health conditions including anxiety/panic attacks (73% vs 59% vs 34% in ≥2, one and no ACEs group respectively; *p* < 0.01), depression (51% vs 30% vs 16%; *p* < 0.01), attention deficit hyperactivity disorder (21% vs 12% vs 7%; *p* = 0.01), and obsessive-compulsive disorder (13% vs 10% vs 3; *p* = 0.03). Those youth with two or more ACEs were significantly more likely to have previously utilized outpatient mental health care (72% vs 59% vs 53%; *p* = 0.01), a previous psychiatric hospitalization (11% vs 4% vs 2%; *p* < 0.01), or have endorsed suicidal ideation (28% vs 17% vs 13%; *p* = 0.01). There were no significant differences in likelihood of a comorbid eating disorder, learning disability, or previous suicide attempt (Table 1). Thirty-six patients (9%) had a comorbid rheumatologic diagnosis with similar proportions across cohorts.

In secondary analyses, we found that 75% of our cohort with co-morbid rheumatologic disease reported having at least one ACE, rates much higher than the general pediatric population [29, 30]. There was no significant association detected between ACEs and presence of rheumatologic disease, although this was an exploratory analysis, and we were likely underpowered to test this association.

We assessed factors such as ACEs and other variables of interest impacting functional disability (FDI score) in patients with chronic pain. Bivariate linear regression indicated that patients with two or more ACEs had higher estimated functional disability (β = 2.28) than those with no ACE exposure however, this association was not statistically significant (*p* = 0.17, Table 3). Similarly, an increase in number of co-morbid rheumatologic conditions was not statistically significant as a predictor for FDI. Apart from that, when WPI, SSS and verbal pain score increased by one point, the estimated average FDI score increased by 0.67, 2.26 and 1.92, respectively (all *p* < 0.05; Table 3). The estimated FDI score was almost 5-points higher in those patients who reported autonomic changes than those without autonomic changes (β = 4.97; *p* < 0.001). Additionally, presence of self- or parent-reported mental health conditions were associated with increased FDI scores in patients, including anxiety (β = 2.38; *p* = 0.04), depression (β = 5.01; *p* < 0.01), obsessive compulsive disorder (β = 7.20; *p* < 0.01) and suicidal ideation (β = 4.26; *p* < 0.01). Additionally, estimated FDI score was, on average, higher by 2.61 and 7.25 points in those who received outpatient mental health care and psychiatric hospitalization, respectively (*p* < 0.05; Table 3).

**Table 3:** Bivariate Linear Regression Model for Functional Disability reported by Youth with Chronic Pain (*N* = 412)

No ACEs	β Estimates	95% Confidence Interval		*P*-value
	Ref	Ref	Ref	–
1 ACE	− 1.02	−3.97	1.92	0.49
≥2 ACEs	2.28	−0.96	5.53	0.17
Verbal pain score(0–10)	1.92	1.55	2.29	<.0001
WPI (0–19)	0.67	0.47	0.87	<.0001
SSS (0–12)	2.26	1.95	2.56	<.0001
Duration of symptoms (months)	−0.03	−0.08	0.01	0.13
Autonomic Changes	4.97	2.32	7.63	<.001
History of Mental Health conditions^¶^	4.62	1.72	7.52	< 0.01
Number of Co-morbid Rheumatologic conditions	−1.54	−4.59	1.51	0.32

Abbreviations: *WPI* Widespread Pain Index (total score ranges from 0 to 19 where higher score indicates more widespread pain), *SSS* Symptom Severity Score (total score ranges from 0 to 12).

^¶^History of mental health conditions includes presence of one or more self- and/or parent- reported cognitive and/or psychological issues including anxiety, depression, OCD, suicidal ideation; or patient who received outpatient or inpatient mental health care

Upon exploring the interaction between ACE exposure and co-morbid rheumatologic conditions among this cohort of youth with chronic pain, each added co-morbid rheumatologic condition was associated with an estimated average increase in FDI score by 8.5 points in those with one ACE (*p* = 0.07), and 11 points in those with 2 or more ACEs (*p* = 0.03), with those patients with no ACEs serving as the reference group.

**Fig. 2:**
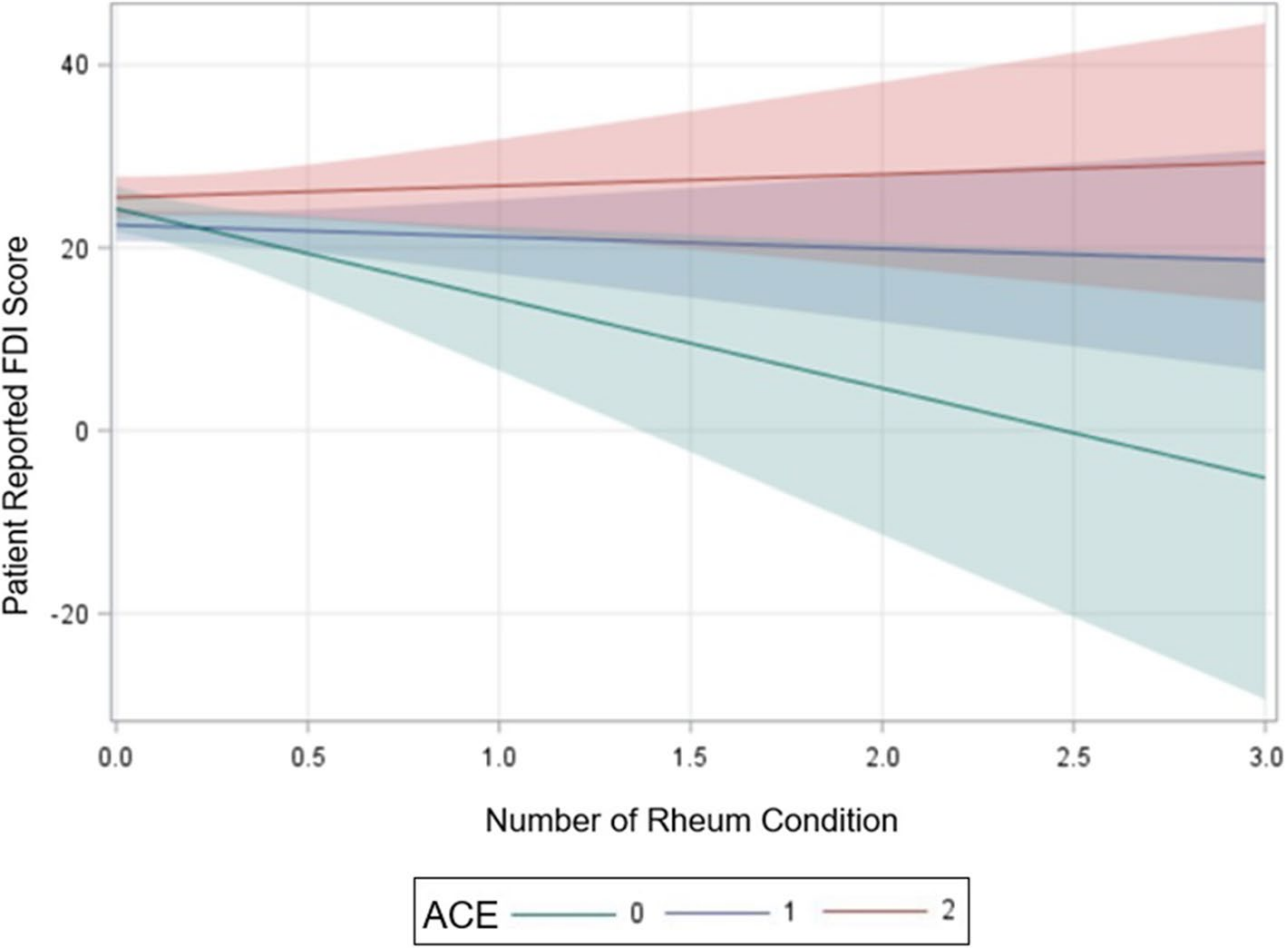
Predicted Values of Patient Reported FDI score for Each Co-morbid Rheumatic Disease Stratified by Level of ACEs Exposure**.** Patient reported FDI score 0–60; Number of Rheum Conditions 0,1,2

Figure 2 illustrates the slope of predicted FDI changes with number of rheumatologic conditions for each level of ACE exposure indicating interaction effect; the shaded area suggests the 95% confidence interval (CI) associated with each slope. Overall, the CI for predicted FDI was in the moderate (FDI 21–29) to severe (FDI ≥30) functional disability range for those with two or more ACEs whereas the CI for those with one ACE lay within the mild (FDI 13–20) to moderate functional disability range. This suggests worse functional disability in those with ≥2 ACEs with a co-morbid rheumatologic condition.

Our multivariable regression model included variables of interest that were found to have significant association with FDI at the individual level to test their independent impact on functional disability after adjusting for other variables of interest. In the multiple linear regression model (Table 4), we found that increases in verbal pain score, symptom severity score, and presence of autonomic changes were independently associated with estimated average increases in FDI score by 1.05, 1.95 and 4.76 points respectively (all *p* < 0.01). The interaction showing worsened functional impairment from rheumatologic disease for those with ACEs was again observed. For each co-morbid rheumatologic condition, estimated increase in average FDI was 6.85 points for those with one ACE (*p* = 0.05), and 8.08 points for those with two or more ACEs (*p* = 0.03), after adjusting for pain score, symptom severity score, autonomic changes, and presence of mental health conditions. In contrast, youth with chronic pain and rheumatic disease without any ACEs, did not have worsened functional disability (β = − 6.71 [− 12.93,-0.49]; *p* = 0.03).

**Table 4:** Multiple Linear Regression Model for Functional Disability reported by Youth with Chronic Pain (*N* = 412)

No ACEs	β Estimates	95% Confidence Interval		*P*-value
	Ref	–	–	–
1 ACE	−2.96	−5.31	−0.61	0.01
≥2 ACE	−1.02	−3.69	1.66	0.46
#Rheumatologic conditions	−6.71	−12.93	−0.49	0.03
1 ACE*#Rheumatologic conditions	6.85	−0.14	13.83	0.05
≥2 ACEs*#Rheumatologic conditions	8.08	0.72	15.44	0.03
Verbal pain score (0–10)	1.05	0.72	1.39	<.0001
Autonomic Change, Yes	4.76	2.67	6.86	<.0001
SSS (0–12)	1.95	1.64	2.27	<.0001
History of Mental Health conditions^¶^, Yes	−0.33	−2.70	2.03	0.78

Abbreviations: #Rheumatologic conditions = number of rheumatologic conditions, *SSS* Symptom Severity Score (total scores range from 0 to 12).

^¶^History of mental health conditions includes presence of one or more cognitive and/or psychological issues including anxiety, depression, OCD, suicidal ideation; or patient who received outpatient or inpatient mental health care

## Discussion

Our study’s findings build upon and further describs the relationship between ACEs and pediatric chronic disease as suggested by Rubinstein et al. [12]. In the most recent survey of the NSCH, just under half of children were estimated to have an ACE, but nearly three-quarters of children with parent-reported arthritis had experienced an ACE. Similarly, in the present study, 76% of youth with chronic pain had experienced at least one ACE, a rate much higher than the general population, demonstrating a strong relationship between ACEs and pediatric chronic pain.

The etiologies of rheumatologic disease and chronic pain are both complex and multifold. However, previous studies have shown that they share many upstream factors such as genetic predisposition and environmental exposures that can increase one’s susceptibility to disease. Rheumatologic disease has also been shown in adults to be associated with chronic stress, although such relationships have not been well described in children. Our results show a rate of comorbidity between chronic pain conditions and rheumatologic disease that is significantly higher than the rate of pediatric rheumatologic disease in the general population, further suggesting upstream factors for these conditions are intertwined [29, 30].

Our study’s findings suggest important implications for prognosis and treatment recommendations for youth with chronic pain and rheumatic disease. For example, our study found that youth with a greater number of ACEs had more functional disability from a co-morbid rheumatic disease. Presence of positive social factors such as a caring adult, involvement in extracurricular activities, and access to robust social services have all been shown to mitigate the impact of ACEs [31–33]. Exploring the applicability of wraparound social services could be of increased importance in optimal treatment for children with both ACEs and rheumatologic disease. Our findings further suggest that inclusion of ACEs in routine screening could help identify those in the general pediatric population who are at higher risk of developing chronic pain before such pain develops.

Untreated chronic pain poses significant risk to a child’s normal growth and development, a concern amplified in children with ACEs. Our results found that while ACEs were not associated with the severity of pain, there was a dose-dependent response where chronic pain youth with ACEs experienced greater difficulty in processing their pain, as reflected by greater functional disability and higher rates of comorbid mental health concerns including depression, anxiety, obsessive compulsive disorder (OCD), and suicidal ideation. This finding suggests that the impact of ACEs centers around how pain is interpreted and processed, rather than the sensation of pain itself. Thereby, pain was more likely to have a permeating effect through other aspects of the lives of children with ACEs than children without ACEs as a risk factor.

These findings present an opportunity for pediatric rheumatologists and general pediatricians alike. There are numerous protective factors that can insulate a child from the toxic effect of ACEs, as described by the guidance released by the AAP Committee on Child Abuse and Neglect [34]. These include involvement in physical activity and extracurricular activities, promoting an internal locus of control, and the presence of a caring and stable adult figure – all aspects of the current standard of care for chronic pain syndromes. Providers with awareness of the links between ACEs and chronic illness (including both pediatric rheumatic disease and chronic pain) can help patients engage in protective activities and refer at-risk youth to a pain specialist earlier in the course of development of symptoms to initiate timely and effective treatment.

We do want to address the limitations of this study, of which there are several. First, there were a low number of the more profound ACEs (household member with substance use disorder, household member attempting suicide, etc.). As such, we were also unable to perform subgroup analyses to explore if these particular ACEs may have larger effect sizes. It is likely that these ACEs associated with greater social stigma were less likely to be volunteered by the family and uncovered during initial clinic psychologic evaluation. This creates an ascertainment bias in our data, however, this bias would point towards the null hypothesis and lead to underappreciation of the significance of ACEs, meaning effects may be even greater than our study found. Similarly, we may not have assessed for all relevant ACEs due to how ACEs were defined and identified for our study [35], suggesting rates could be even higher in our population than we report. We also did not have family income data and had to infer based on zip code level data. It is possible that our current metrics are insufficiently precise to understand the full relationship between income, ACEs, and chronic pain. We find it notable that our cohort was predominantly White, non-Hispanic, and of higher SES. This limits the generalizability of our findings but also represents, we hypothesize, the limited access some youth with chronic pain have to care in an interdisciplinary chronic pain clinic. This warrants further investigation. Additionally, survey data and parent-reported information was not collected in a way that allows us to ascertain which caregiver/parent completed the measures and there may be ascertainment biases that we are unaware of. Finally, while there was a clear relationship between chronic pain and other rheumatologic disease, we were inadequately powered to determine if this was driven by any particular rheumatologic diagnosis and we could not explicitly determine the causality or directionality of the relationship between ACEs, chronic pain or rheumatic disease.

However, our study highlights how ACEs may influence clinical symptomatology among a chronic pain population. Our findings are hypothesis generating and justify the need for a larger multicenter prospective study with a standardized assessment of ACEs to fully elucidate the interplay between ACEs, chronic pain, and rheumatic disease. Nonetheless, our findings highlight a previously underappreciated importance of directly asking patients about all potential ACEs to better understand their effects on chronic pain, as well as chronic illness more broadly, and to help inform and guide future treatment protocols that better incorporate the social context and the child’s previous traumas in the management of chronic pain.

## Conclusions

We examined the prevalence of ACEs among pediatric patients with chronic pain and rheumatologic diseases, and its impact on clinical symptomology, in a cohort of patients presenting to our rheumatology pain subspecialty clinic. The results indicate that children with chronic pain and/or rheumatologic diseases who are exposed to ACEs are at increased risk of worse functional disability, greater pain-related symptomatology, and a higher burden of co-morbid mental health conditions. Therefore, children with pain conditions should be systematically evaluated for exposure to ACEs as a part of early intervention to mitigate the risk of developing chronic pain with severe features. Our patient population was also markedly different from the general pediatric population in terms of race/ethnicity, gender, and socioeconomic background suggesting there may be sociocultural factors affecting patient presentation to care in a pain clinic that should be examined further. Future studies focusing on systematic screening and assessment of ACEs can help to determine the definite burden of ACEs in the pediatric population with musculoskeletal pain and/or rheumatic disease which will be further helpful to identify those youth with ACEs at higher risk of developing chronic pain before such pain develops and optimize treatment modalities in a patient-centered manner.

## Data Availability

All data generated or analysed during this study are included in this published article [and its supplementary information files]. The datasets generated and/or analysed during the current study are not publicly available due protection of patient privacy but are available from the corresponding author on reasonable request.

## Abbreviations

ACEs: Adverse childhood experiences
FDI: Functional Disability Inventory
CNS: Central nervous system
NSCH: National Survey of Children’s Health
HPA: Hypothalamic-pituitary-adrenal
RedCap: Research Electronic Data Capture
PROs: Patient reported outcomes
SSS: Symptom severity score
WPI: Widespread pain index
IQR: Interquartile range
CI: Confidence interval
OCD: Obsessive compulsive disorder
AAP: American Academy of Pediatrics
